# Pulse Oximetry Screening for Detecting Critical Congenital Heart Disease in Neonates

**DOI:** 10.7759/cureus.32852

**Published:** 2022-12-23

**Authors:** Deepshikha Jain, Manish Jain, Yashwant Lamture

**Affiliations:** 1 Pediatrics, Himalayan Institute of Medical Sciences, Swami Rama Himalayan University, Dehradun, IND; 2 Pediatrics, Mahatma Gandhi Institute of Medical Sciences, Sevagram, IND; 3 Surgery, Datta Meghe Institute of Medical Sciences, Wardha, IND

**Keywords:** muconium aspiration, cyanosis, hypoxemia, echocardiography, oxygen saturation

## Abstract

Background

Congenital heart disease (CHD) leads to significant morbidity in the neonatal population and is a crucial disorder behind early infancy death rates. Most have a critical congenital heart defect (Cr CHD) out of all the heart defects found in babies. A subgroup of cardiac anomalies needs surgery or catheter intervention during the neonatal period. Pulse oximetry is a good screening tool to detect cr CHD in neonates. This study aims to assess the effectiveness of pulse oximetry as a screening tool in a rural setting.

Methods

This was a hospital-based prospective observational study. All consecutively born neonates whose parents consented to the study were subjected to pulse oximetry on all four limbs. Their peripheral arterial oxygen saturation was measured on days one, two, and three of life, starting four hours after birth. Babies detected with cyanotic congenital heart disease (CCHD) before birth are not a part of this study. All those with arterial oxygen saturation of less than 95% or a difference of saturation of more than 3% in the upper and lower limbs were considered suspects for Cr CHD and subjected to echocardiography.

Results

Among 5874 neonates studied, researchers found 164 babies to have significant hypoxemia on pulse oximetry, and 44 CHD were detected on echocardiography within the first three days of life (positive predictive value (PPV) 12.2%). The physician referred all of them to a higher center before further delay. Thirty-four babies with other congenital heart diseases were found using pulse oximetry examination. Also, 108 cases of hypoxemia due to other causes were found and monitored.

Conclusion

Critical congenital heart diseases are a significant cause of death among neonates and require early diagnosis and emergent medical and surgical management. They are associated with hypoxemia, and this principle can be used to screen them using a pulse oximeter.

## Introduction

Congenital heart disease (CHD) causes significant morbidity in the neonatal population, comprising 24% of all birth defects, and is behind early infancy death rates [1]. Around 25% have a critical congenital heart defect (Cr HD) out of all the heart defects found in babies. A subgroup of cardiac anomalies needs surgery or catheter intervention in the neonatal period [2]. The birth prevalence for cyanotic congenital heart disease (CCHD) is 2-3/1000 live births globally; in India, it is around 8:1000 [3,4]. According to a report, CHD accounts for 10% of India's infant mortality [5]. Congenital heart disease leads to approximately 40% of infant death worldwide [6].

The persistence of fetal circulation after birth in a few infants can mask the clinical presentation of CHD. Hence clinicians may discharge them considering normal while harboring dangerous heart anomalies [7]. Heart disease can cause feeding problems, respiratory distress, growth impairment, and end-organ damage to vital organs such as the kidneys, the brain, and the eyes. The neonate can quickly end up with congestive cardiac failure, cardiogenic shock, and sudden death [8]. With early diagnosis, however, most infants with Cr CHD can benefit from successful surgical repair or palliation now available with advances in pediatric cardiology and cardiac surgery [9-11]. Screening during pregnancy for congenital anomalies is commonly performed in most of the world and includes clinical examination, blood investigations, anomaly scans, amniocentesis, and chromosomal assessments [12].

However, essential screening tools such as fetal echocardiography are not readily available and can be used only in tertiary care hospitals in developing nations [13]. Clinical examination for heart diseases is cumbersome, often spurious, and requires expertise. Various studies have proved that diagnostic modalities like clinical examination, electrocardiogram (ECG), X-ray, antenatal ultrasonography, or echocardiography are neither practical nor feasible as screening tools in rural and tribal areas [14].

All neonates with CHD have some degree of hypoxemia which can be used for screening them via a pulse oximeter [15-17]. As the studies on pulse oximetry to detect CHD are sparse, this study was undertaken to know the feasibility and significance of it in detecting CHD in neonates.

## Materials and methods

The present study was undertaken in the department of pediatrics, Mahatma Gandhi Institute of Medical Sciences (MGIMS), Wardha, India, after getting approval from the MGIMS Institutional Ethics Committee (IEC) (approval no. MGIMS/IEC/PED/177/2012). It was a hospital-based prospective observational study. All neonates born and admitted to the neonatal intensive care unit (NICU) or postnatal ward were included in the study. Pulse oximetry was done on the neonates' upper and lower limbs. All those with arterial oxygen saturation <95 % or a difference of saturation of more than 3% in the upper and lower limbs were considered suspects for CCHD and subjected to two-dimensional (2D) echocardiography.

Inclusion criteria and exclusion criteria

All neonates born in our hospital were included. Parents gave consent for pulse oximetry and 2D echocardiography (if required). Neonates with a prenatal sonographic diagnosis of duct-dependent circulation and those whose patients did not give consent were excluded. Babies with detected CCHD before birth were not a part of this study.

Methodology and statistical data analysis

A predesigned written informed consent was given to parents. Starting four hours after birth and ensuring that the baby's limbs were warm, researchers obtained pulse oximetry readings on both upper and lower limbs.

A reading > 95% was considered a test negative. Researchers considered a value < 90 % in all limbs or a difference of upper and lower limb values > 3% for testing positive; neonates were subjected to a 2D echocardiography by a pediatric cardiologist. For 90% to 95% saturation, the neonate was re-evaluated after oxygenation for six hours. Some neonates were found to have an improvement in saturation to a level > 95 %, while others did not. All of them were subjected to a 2D echocardiography. Cases with hypoxemia were subjected to a detailed second clinical examination using an examination of peripheral pulses, cyanosis, careful palpation, percussion and auscultation, chest radiograph, pulse oximetry, and echocardiography. Patients were followed with pulse oximetry on day two and day three of life before discharging them. 

Statistical data analysis was done using descriptive and inferential statistics. Inferential statistics helps develop a good understanding of the population data by analyzing the samples obtained. It helps make generalizations about the population using various analytical tests and tools. Here researchers used an accuracy test such as the positive predictive value (PPV). The software used in the research was SPSS version 17.0 (IBM Corp., Armonk, NY, USA) and Graph Pad Prism 5 (GraphPad Software, San Diego, CA USA).

## Results

**Figure 1:**
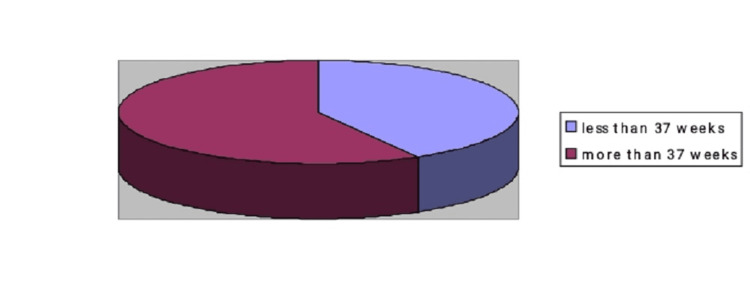
Gestational age of Cr CHD Amongst those diagnosed as having critical congenital heart disease, five (41.67%) were < 37 weeks by gestation, and the remaining seven (58.33%) were >37 weeks. Cr CHD: Critical congenital heart disease

**Figure 2:**
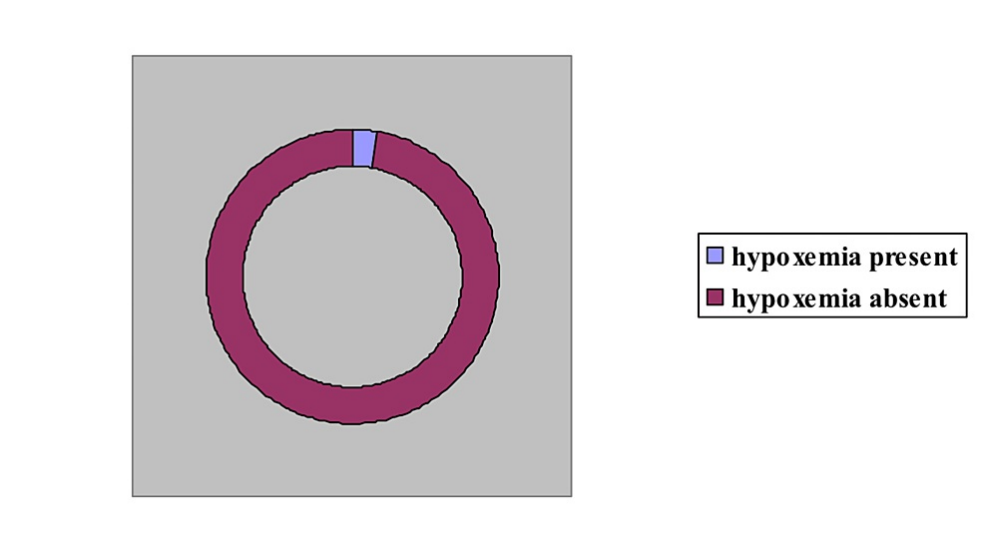
Hypoxemic infants detected on pulse oximetry Out of 5874 neonates, 164 (2.79%) were positive for hypoxemia on pulse oximetry.

**Figure 3:**
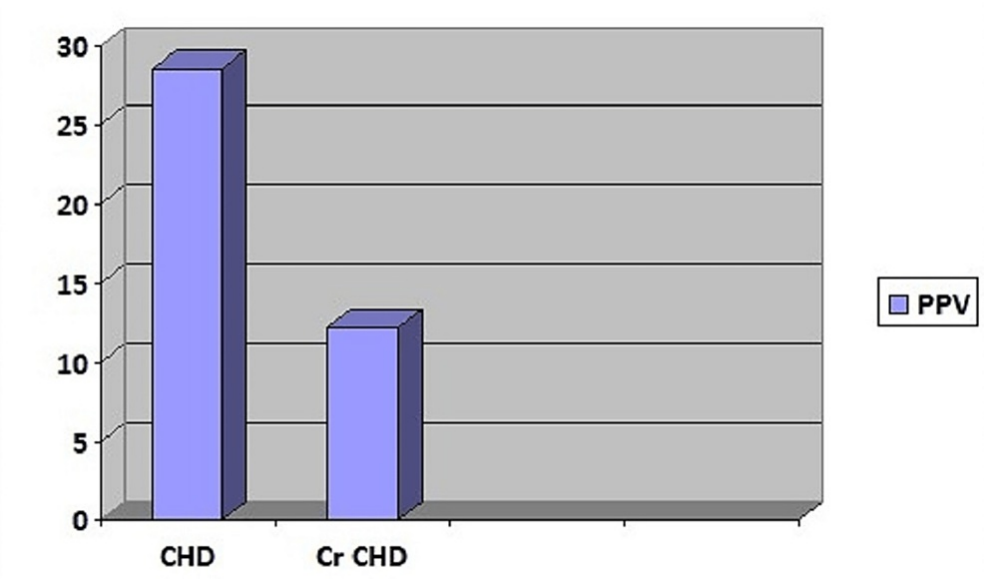
Positive predictive value for congenital and critical heart disease PPV: Positive predictive value, Cr CHD: Critical congenital heart disease, CHD: Congenital heart disease

Among the 5874 neonates studied, 164 had hypoxemia on pulse oximetry. Forty-four had CCHD on echocardiography within the first three days of life (positive predictive value 12.2%). All of them were referred to a higher center before further delay (Figures 1, 2, 3).

Forty-four children affected with CHD were further subdivided into 12 major cardiac defects and 32 minor cardiac defects. Thirty-four babies with other congenital heart diseases were found using pulse oximetry examination (Table 1). The cardiac defects found in these 12 cases of Cr CHD are listed in Table 2.

**Table 1:** Gender distribution in detected congenital heart disease neonates Out of the study population of 5874 neonates, 51.33% were male and 48.6% were female.

Gender	Non-critical congenital heart disease	Critical congenital heart disease
Male	21	6
Female	13	6
Total	34	12
M: F Ratio	1.61:1	1:1

**Table 2:** Major or critical congenital heart diseases detected

Critical or major CHD	
Pulmonary atresia and stenosis	5
Transposition of great arteries	2
Total anomalous pulmonary venous return	3
Transposition of great arteries with a ventricular septal defect	1
Functional univentricular heart	1
Total	12

## Discussion

A study in India conducted by Vaidyanathan et al. introduced using a pulse oximeter to diagnose CHD [18]. In their research, the clinicians and staff nurses did simultaneous clinical examinations and pulse oximetry. They found that the diagnostic accuracy of pulse oximetry, even with physical examination, was low (sensitivity <20%) [18]. Pulse oximetry has been evaluated in different clinical scenarios and is accepted as a routine screening method for CHD in the USA. Similar studies are being conducted all across Europe because of the benefits and ease of use [19,20].

The present study was conducted with even fewer resources. Five neonates out of 5884 (0.08%) had a prenatal diagnosis of duct-dependent circulation on antenatal sonography, indicating a poor screening rate. Among those diagnosed as having Cr CHD, there were six (50%) male children and six (50%) female children (as seen above in Table 1). No gender was considered a risk factor for one-year survival with CHD in a study by Oster et al. [21].

The majority of the neonates were born full-term (73%). Both critical and non-critical CHD were noticed in full-term neonates, i.e., 58.3% and 85.29%, respectively (as seen above in Figure 1). Only five parents (0.08%) did not consent to the study, while the remaining parents were keen to participate and found pulse oximetry safe for their baby. Out of the total 164 2D echocardiographs, 120 did not show any cardiac defect. The high number of false positive cases was because we performed pulse oximetry before 24 hours of life. Many studies noticed that false positives are far less when screening is done between 24 to 48 hours of life [19,20]. Contrary to this, we did not find any new cases of CHD on day two.

Due to resource limitations, on-spot 2D echocardiography could not be done except for a few cases. All patients with Cr CHD were referred to a higher center for further management. Among those diagnosed with Cr CHD, one case with tetralogy of Fallot was readmitted on day seven of life and succumbed two days later. It is a routine practice in our hospital to follow all discharged neonates at 15 days of life and subsequently at six weeks for vaccination, except for high-risk neonates who are followed every 15 days. No other cases labeled as Cr CHD were returned to our hospital.

**Figure 4:**
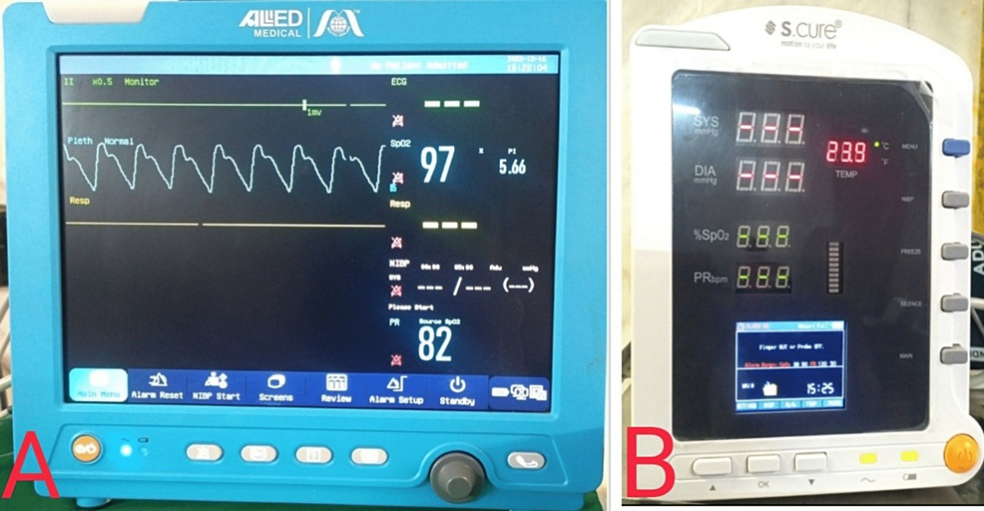
Images of pulse oximeters available in the market A: Pulse oximeter marketed and manufactured by Allied Medical (Allied Medical Ltd. Gurugram, HR, India); B: Pulse oximeter marketed and manufactured by S.Cure (S.Cure Medserv Pvt. Ltd., New Delhi, India)

Out of 5874 neonates, 164 (2.79%) were positive for hypoxemia on pulse oximetry (as seen above in Figure 2). Many brands of pulse oximeters are available in the market, namely Microtek (Microtek International Inc., Hsinchu, Taiwan), L&T (L&T Technology Services, Vadodara, GJ, India), Dr. Morepen (Morepen Ltd. New Delhi, India), Allied Medical (Allied Medical Ltd. Gurugram, HR, India), S Cure (S.Cure Medserv Pvt. Ltd., New Delhi, India), etc., with a price range of 500 INR to 20,000 INR (Figure 4).Those with profound hypoxemia (saturation of peripheral oxygen (SPO2) <90%) were subjected to 2D echocardiography. Around 28.5% of neonates were positive for CHD. In the group where the difference in upper and lower extremity saturation was > 3%, three out of six (50%) were cases of Cr CHD. One hundred forty-four neonates had pulse oximetry values between 90% to 95%. On a repeat observation after six hours, hypoxemia persisted in 78 (54.16%) neonates. Five (6.4%) out of these 78 neonates were positive for Cr CHD.

No CHD was found among those with improved pulse oximetry values (after six hours). Amongst false positives, there was severe birth asphyxia in nine (7.5%) cases, meconium aspiration syndrome in 14 (11.66%) cases, sepsis in 67 (55.8%) cases, persistent pulmonary hypertension in 16 (13.3%) cases, pneumothorax in two (1.66%) cases, and 12 (10%) patients were normal. During the screening, 32 cases of other congenital heart diseases were documented (Table 3).

**Table 3:** Types of other congenital heart diseases observed VSD: Ventricular septal defect, PDA: Patent ductus arteriosus

Congenital heart disease	Size	Number
Atrial septal defect (ASD)	Small	
	Moderate	3
	Large	
Ventricular septal defect (VSD)	Small	12
	Moderate	4
	Large	3
(PDA)	Small	8
	Moderate	
	Large	1
VSD with PDA		1
Total		32

Among these 32 cases, 16 (50%) were positive for hypoxemia on pulse oximetry. Of these 16 cases, 25% had significant lesions, and the remaining 12 had minor lesions. Our study found the predictive value of a positive test for Cr CHD was 12.2% (as seen above in Figure 3). The study by Reid et al. reported a PPV of 25.93% for Cr CHD [19]. We used both pre-ductal as well as post-ductal saturation values. In the study by Reid et al., it is proven that some forms of CHDs with the predominant right to left shunting at the ductal level may be missed when researchers took only pre-ductal measurements. However, reports with good follow-up data, higher echocardiography coverage, and better antenatal ultrasonography diagnostic value have suggested sensitivity, specificity, and positive and negative predictive values at 77.78%, 99.90%, 25.93%, and 99.99%, respectively. In the light of knowledge that the actual number of true negative and false negative cases is not known precisely, the test's sensitivity, specificity, and negative predictive value cannot be commented upon, making it a significant limitation of the study.

In our study within a tertiary-based rural hospital setup, the incidence of CHD was two per 1000 live births. Out of the total of 164 echocardiograms done, 120 did not show any cardiac defect. The high number of false positive cases was because we performed pulse oximetry before 24 hours of life. Many studies noticed that false positives are far less when screening is done between 24 to 48 hours of life [18-20].

Contrary to this, we did not find any new cases of CHD on day two or three. Due to resource limitations, on-spot echocardiograms could not be done except for a few cases. All patients with a Cr CHD were referred to a higher cardiac center for further management. Among those diagnosed with Cr CHD, one case with tetralogy of Fallot was readmitted on day seven of life and succumbed two days later. It is a routine practice in our hospital to follow all discharged neonates at 15 days of life and subsequently at six weeks for vaccination, except high-risk neonates who are followed every 15 days. No other cases labeled as Cr CHD were returned to our hospital. No cases of left heart obstructive lesions could be found. Our study found the predictive value of a positive test for Cr CHD was 12.2%.

Our study's predictive value of a positive test for CHD was 28.5%. The low number of Cr CHDs may be the explanation behind the higher PPV for congenital disease compared to that of critical congenital heart disease. The study's results reveal the inherent limitations of clinical screening for Cr CHD in newborns immediately after birth, especially in the context of limited resource environments. It is not feasible to do an echocardiogram of every neonate, especially in areas with a high birth rate like India.

Plana et al. concluded that pulse oximetry has good diagnostic accuracy with moderate sensitivity for identifying Cr CHD with meager false‐positive rates [22]. They recommend it as a screening tool in asymptomatic newborns before discharge from the hospital. These are similar to the present study's recommendations.

The actual number of true negative and false negative cases has yet to be discovered precisely. The test's sensitivity, specificity, and negative predictive value cannot be commented upon, making it a significant study limitation. However, reports with good follow-up data, higher echocardiography coverage, and better antenatal ultrasonography diagnostic value have suggested sensitivity, specificity, and negative predictive value to be 77.78%, 99.90%, 25.93%, and 99.99%, respectively [18].

The Cr CHD incidence at our tertiary rural hospital was two per 1000 live births. The incidence of CHD was found to be 7.8 per 1000 live births. Thus, in a resource-limited environment, pulse oximetry screening for Cr CHD contributes to its early diagnosis. The added advantage is a diagnosis of other morbidities and its utilization in further monitoring those with hypoxia.

Limitations

Since the study was conducted in a rural tertiary care hospital, the study's findings cannot be generalized to the population as a whole. It is the inherent limitation of the hospital-based study compared to community-based research. Due to resource constraints, doing a 2D echocardiogram of each neonate was not feasible in our study.

## Conclusions

The study's sampling was done where all consecutive neonates whose parents gave consent were included to avoid selection bias. This study provides a fair presentation of the patient population of the rural tertiary-based hospital in India. As there is a lack of data regarding the magnitude of CHD in India, this study can serve as baseline data to compare and follow for further studies.

Pulse oximetry is an effective adjunct to antenatal ultrasonography and appropriate clinical examination in diagnosing Cr CHD, even in a resource-limited environment. It facilitates early referral and treatment of patients diagnosed with Cr CHD at a center equipped for their care. Resource generation in the form of pulse oximeters and hospital staff trained to do pulse oximetry is feasible in a developing country like India.
